# Photocathode functionalized with a molecular cobalt catalyst for selective carbon dioxide reduction in water

**DOI:** 10.1038/s41467-020-17125-4

**Published:** 2020-07-13

**Authors:** Palas Baran Pati, Ruwen Wang, Etienne Boutin, Stéphane Diring, Stéphane Jobic, Nicolas Barreau, Fabrice Odobel, Marc Robert

**Affiliations:** 1 Université de Nantes, CNRS, CEISAM UMR 6230, F-44000 Nantes, France; 2 Université de Paris, Laboratoire d’Electrochimie Moléculaire, CNRS, F-75006 Paris, France; 3 Université de Nantes, CNRS, Institut des Matériaux Jean Rouxel, IMN, F-44000 Nantes, France; 4 Institut Universitaire de France (IUF), F-75005 Paris, France

**Keywords:** Chemistry, Catalysis, Electrocatalysis, Heterogeneous catalysis, Homogeneous catalysis

## Abstract

Artificial photosynthesis is a vibrant field of research aiming at converting abundant, low energy molecules such as water, nitrogen or carbon dioxide into fuels or useful chemicals by means of solar energy input. Photo-electrochemical reduction of carbon dioxide is an appealing strategy, aiming at reducing the greenhouse gas into valuable products such as carbon monoxide at low or without bias voltage. Yet, in such configuration, there is no catalytic system able to produce carbon monoxide selectively in aqueous media with high activity, and using earth-abundant molecular catalyst. Upon associating a p-type Cu(In,Ga)Se_2_ semi-conductor with cobalt quaterpyridine complex, we herein report a photocathode complying with the aforementioned requirements. Pure carbon dioxide dissolved in aqueous solution (pH 6.8) is converted to carbon monoxide under visible light illumination with partial current density above 3 mA cm^−2^ and 97% selectivity, showing good stability over time.

## Abstract (web summary)

Photo-electrochemical reduction of carbon dioxide is a promising strategy to derive valuable products. Here, the authors show a photocathode able to produce carbon monoxide selectively in aqueous media with high activity using earth-abundant molecular catalyst.

## Introduction

Artificial photosynthesis is an important research area, since it represents one of the long-term credible solutions to reduce our dependence toward fossil fuels, and to produce storable chemical fuels or raw materials for chemical industry with a low carbon footprint. Directly inspired from natural photosynthesis, which consists of transforming atmospheric CO_2_ into biomass with sunlight, solar-driven conversion of CO_2_ into CO is a key transformation for artificial photosynthesis. Indeed, CO is a strategic building block for chemical industry, since it can be transformed into any liquid carbon-based hydrocarbons by Fischer–Tropsch process or into methanol and acetic acid by Cativa–Monsanto process, for which the markets and the infrastructures are already in place. Namely, there are several strategies to achieve CO_2_ photoinduced reduction. Homogeneous photochemistry with molecular catalysis usually offers good selectivity, but catalyst reusability and coupling the reduction process with a useful and benign oxidation reaction remain a challenge although indispensable for practical applications^1–3^. Heterogeneous catalysis at an electrode offers the opportunity to split oxidative and reductive processes into two different compartments through electrochemistry. An approach closely inspired by homogeneous photochemistry is to graft both photosensitizer (PS) and catalyst at an electrode, reproducing conditions of photochemistry at the interface, then replacing the sacrificial electron donor by an electrode. This strategy has been proven to be efficient in terms of selectivity and applied potential, but remains subject to low current density (typical photocurrent density being well below 100 μA/cm^2^)^4–6^, owing to dominant charge recombination, notably between the molecular photocatalytic system and the holes in the semiconductor (SC). Another approach, inspired by photovoltaic (PV) cells, consists of positioning the catalyst at the interface between a p-type SC electrode and the electrolyte. This strategy has been the object of interest in the 1980s^7–9^ and has attracted renewed attention over the last 10 years, leading to stimulating results^10–14^. Studies in water are scarce, usually involving the use of precious metal complexes as catalysts^11,15^ and/or necessitating large overpotential^8^. More recently, photocathodes displaying a buried junction have emerged^16–20^. In this configuration, a narrow-bandgap p-type SC, that is often subject to photocorrosion^21^, is protected by a wide-bandgap n-type SC in order to get longer lifetime and form a complete p–n junction. This protecting layer, usually constituted of several wide-bandgap SCs, also offers an interesting platform for grafting catalyst. In early reports from Grätzel, Mayer et al., good current densities were obtained with high selectivity for CO (>80%), but these studies were performed in organic solvent, with a precious Re complex catalyst (first in solution^16^ and then grafted onto the electrode^17^), and they required rather negative potentials (ca. −1.75 V vs. Fc^+^/Fc). Recently, a hematite (Fe_2_O_3_) photocathode co-doped with nitrogen and zinc has been reported^18^. The surface of the iron oxide was coated with a layer of TiO_2_ and a Ru complex was then electropolymerized on top. In aqueous electrolyte and under simulated sunlight, the system was able to achieve a selectivity of 63% for formate or 66% for CO, depending on the catalyst used. With one of these catalysts, total photocurrent density of 0.15 mA cm^−2^ (*j*_HCOO_− = 0.094 mA cm^−2^, *j*_CO_ = 0.05 mA cm^−2^) under an applied bias of +0.1 V vs. RHE (200 mV underpotential) was maintained for several hours of operation. A junction made of p- and n-type silicon nanowires (p-Si and n-Si, respectively) and covered by n-GaN was also reported and associated with a molecular assembly composed of a Ru tris–bipyridine dye and a Ru bipyridine complex acting respectively as a light harvester and a CO_2_ reduction catalyst^19^. In neutral aqueous medium and under sunlight irradiation, this multiple-absorber system generated formate with Faradaic efficiency (FE) in the range 35–65% and with a maximum partial photocurrent density for formate of 0.72 mA cm^−2^ at −0.25 V vs. RHE. Remarkably, the current density was stable for more than 20 h of operation, but again precious metal complex was used, and the applied potential remains rather negative for a system containing two light absorbers assembled in series. Eventually, Reisner et al. developed a buried junction photocathode featuring an earth-abundant metal catalyst. A p-Si SC was covered with a film of TiO_2_ nanoparticles on which bis-terpyridine cobalt complex was anchored via phosphonic acid groups^20^. However, in water, partial photocurrent densities remained low (*j*_CO_ = 0.018 mA cm^−2^ and *j*_HCOO_− = 0.024 mA cm^−2^) at 0 V vs. RHE in neutral medium (0.1 M KHCO_3_), with selectivities of 16 and 21% for CO and formate, respectively. In other words, no photocathode made of earth-abundant molecular catalyst is yet able to present concomitantly, a good selectively for CO or formate, a near-zero overpotential, and large current densities when working in aqueous solution.

Here, we report a photocathode based on Cu(In,Ga)Se_2_ (CIGS) SC and cobalt quaterpyridine catalyst that complies with all of these requirements. This study is also the first report of CIGS SC as a constituent of a molecular-based photocathode for CO_2_ reduction in water. Our step-by-step photocathode elaboration starting from electrochemical (EC) configuration, then PV + EC, and eventually photo-EC (PEC) configuration is detailed.

## Results

### (Photo)-electrode preparation and characterization

**Fig. 1:**
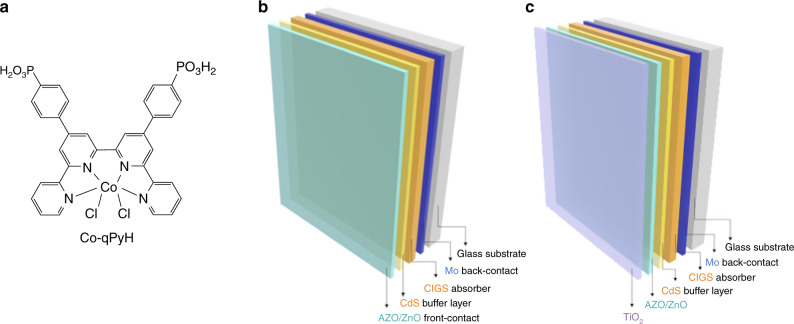
Electrode components. Structure of the **Co-qPyH** molecular catalyst bearing phosphonic acid functions (**a**). Schematic view of the CIGS-based layered material used in PV conditions (**b**) and in PEC conditions (**c**). Additional layers to CIGS are molybdenum (Mo), cadmium sulfide (CdS), aluminum-doped zinc oxide (AZO/ZnO), and titanium dioxide (TiO_2_).

Cobalt quaterpyridine, aside from including an earth-abundant metal, is one of the most efficient CO_2_-to-CO reduction molecular catalyst, operating at overpotential as low as ca. 350 mV in aqueous neutral conditions^22^. Association with a buried p–n junction able to produce this range of photovoltage should thus allow to work near or under thermodynamic potential for CO_2_-to-CO conversion. Here, we prepared the cobalt complex with planar tetradentate ligand 2,2′:6′,2′′:6′′,2′′′-quaterpyridine (**Co-qPyH**, Fig. 1), whose parent compound is known to be an efficient EC but also photochemical CO_2_ reduction catalyst, able to operate in aqueous media with good activity and stability^22–25^. The catalyst is substituted with two phosphonic acid groups allowing for grafting on the surface of a metal oxide layer. Phosphonic acid proved to be among the most stable binding groups on metal oxide surfaces, provided pH range (<7) is respected^26–28^.

**Fig. 2:**
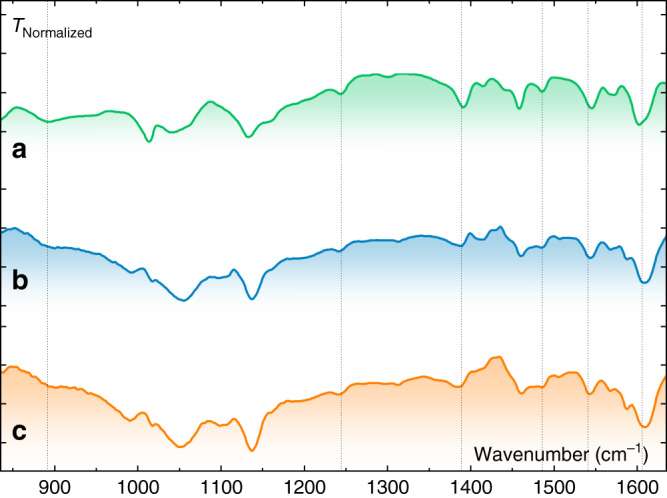
ATR-IR spectrum of Co-qPyH complex catalyst. From a powder (**a**), deposited on **m-TiO**_**2**_ before (**b**) and after (**c**) 1 h of electrolysis performed in 0.1 M KHCO_3_ electrolyte saturated with CO_2_ (pH 6.8) at −0.56 V vs. RHE.

**Co-qPyH** was synthesized by the route described in the “Methods” section and was carefully characterized (Supplementary Fig. 1). This complex exhibits only a weak absorbance in the visible spectrum and therefore leaves the electrode almost transparent to most of the incoming solar irradiation. Flat TiO_2_ (**f-TiO**_**2**_) and mesoporous TiO_2_ (**m-TiO**_**2**_) were used as both grafting surface and protecting top layer. After deposition of this oxide on FTO, Mott–Schottky analysis revealed a donor density (*N*_D_) of 6.3 × 10^20^ cm^−3^ in the conduction band (Supplementary Fig. 2), suitable for an electron-transporting layer. Ultraviolet–visible (UV–vis) absorption spectra were recorded on **m-TiO**_**2**_|**FTO** surface before and after catalyst grafting upon a soaking step (Supplementary Fig. 3), which confirmed the modification of the semiconducting surface. Upon attenuated total reflectance—infrared spectroscopy (ATR-IR) analysis, resemblance in fingerprint regions between **Co-qPyH** powder and **Co-qPyH**-loaded **m-TiO**_**2**_ electrode (**Co-qPyH**|**m-TiO**_**2**_) further suggests that the catalyst is grafted on the surface (Fig. 2). The bands at ∼1390, ∼1490, ∼1546, and ∼1601 cm^−1^ assigned for aromatic ring stretching vibration corroborate structural preservation of the catalyst upon immobilization.

**Fig. 3:**
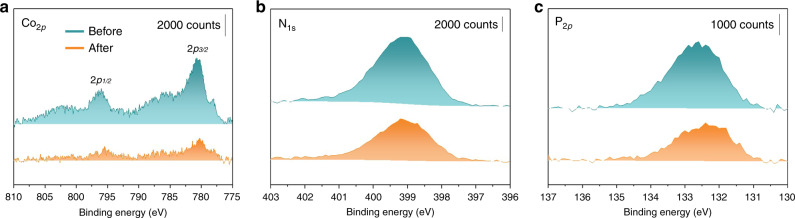
XPS analysis of the Co-qPyH|m-TiO_2_ electrode. Before (blue trace) and after (orange trace) 2 h of electrolysis at −0.51 V vs. RHE in 0.1 M KHCO_3_ electrolyte saturated with CO_2_ (pH = 6.8). Data show binding energies for Co_2p_ (**a**), N_1s_ (**b**), and P_2p_ (**c**).

On the other hand, the disappearance of a band near 893 cm^−1^ (P–OH) of **Co-qPyH** after surface attachment along with the presence of the band at 1243 cm^−1^ (P = O stretching) suggests typical bidentate binding of phosphonate on TiO_2_ surface^26–28^, with P = O functional group not involved in the linkage. XPS analyses (Fig. 3, blue) show a signal for P_2p_ that is characteristic of phosphate (132.5 eV)^17,20^, a N_1s_ signal characteristic of a pyridinic nitrogen complexing a cobalt atom in a quaterpyridine ligand (399.5 eV)^22^, and that of Co_2p_ indicative of a Co^2+^ oxidation state (main peak at 780 eV, secondary peak at 796 eV, and two shoulders at 785 and 803 eV, respectively), confirming the presence of the catalyst. Complementary EDX-mapping experiment (Supplementary Fig. 4) shows a consistent atomic ratio of 2:1 between phosphate and cobalt, definitively proving the presence of the catalyst and confirming its intactness after grafting. Mapping of Co element revealed the absence of cobalt aggregate, suggesting a homogeneous distribution of the catalyst on the overall surface. Inductively coupled plasma optical emission spectrometry (ICP–OES) measurements after catalyst dissolution (acidic treatment), indicate catalyst loading of 29 ± 4 nmol cm^−2^ on **m-TiO**_**2**_ film and 3 ± 1 nmol cm^−2^ in **f-TiO**_**2**_ film (see “Methods” for details). This concentration is about one-third of previously reported data (85 nmol cm^−2^ for a Re complex with a similar anchoring group on mesoporous TiO_2_)^17^, leaving room for improvement.

### EC reduction of CO_2_

**Fig. 4:**
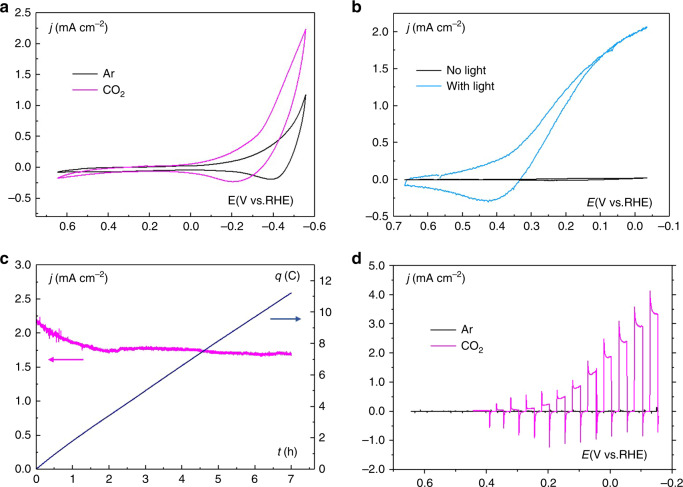
Electrochemical and photoelectrochemical data in various configurations. **a** CV in EC conditions at a **Co-qPyH**|**m-TiO**_**2**_ electrode in 0.1 M KHCO_3_, saturated with argon (black) or CO_2_ (magenta). Scan rate was 20 mV s^−1^. **b** CV in PV + EC conditions at a **Co-qPyH**|**m-TiO**_**2**_ electrode in 0.1 M KHCO_3_ saturated with CO_2_, connected to CIGS solar cells without (black) and with light illumination (blue). Scan rate was 20 mV s^−1^. **c** Long-term electrolysis in PV + EC conditions at a **Co-qPyH**|**m-TiO**_**2**_ electrode in 0.5 M KHCO_3_ saturated with CO_2_ (pH 7.2), connected to an external irradiated CIGS solar cells polarized at −0.03 V vs. RHE with continuous bubbling during electrolysis. **d** LSV in PEC conditions at a **Co-qPyH**|**f-TiO**_**2**_|**CIGS** electrode in 0.1 M phosphate buffer saturated with argon (pH 6.8, black) or 0.1 M KHCO_3_ electrolyte saturated with CO_2_ (pH 6.8, magenta) under chopped light irradiation. Scan rate was 5 mV s^−1^.

The catalytic activity of **Co-qPyH**|**m-TiO**_**2**_ electrode was first explored by cyclic voltammetry (CV). In 0.1 M KHCO_3_ aqueous solution saturated with CO_2_ (pH = 6.8), catalytic current was observed with an onset potential at ca. −0.3 V vs. RHE (Fig. 4a). Long-term electrolysis at −0.51 V vs. RHE (2 h) led to an average catalytic current of 1.2 mA cm^−2^ (Supplementary Fig. 5), and gas chromatography (GC) analysis of the products indicated formation of CO with 80% selectivity and 63% FE along with H_2_ as the by-product (entry 1, Table 1). The current density, after reaching a steady-state value in the first 15 min, remained stable for 2 h without significant change. Electrolysis performed under argon in the same conditions of pH (0.1 M phosphate buffer) and potential did not yield any CO (entry 2, Table 1). An ATR analysis of the electrode before and after 1 h of electrolysis in 0.1 M KHCO_3_ saturated with CO_2_ (pH 6.8) at −0.56 V vs. RHE (Fig. 2) showed good stability of the catalyst and the grafting since characteristic signals remained almost unchanged. XPS analysis of the electrode before and after a longer (2 h) electrolysis in 0.1 M KHCO_3_ saturated with CO_2_ (pH 6.8) at −0.51 V vs. RHE (Fig. 3) confirmed such stability even if the intensity of the signal was slightly dampened. This signal-intensity decrease is pointing toward slow catalyst desorption rather than catalyst decomposition. This is most likely due to the fact that working pH is close to the upper limit for phosphonate group stability^27^. Nevertheless, the above results indicate that the cobalt quaterpyridine complex kept its catalytic activity after functionalization with phosphonic acid and immobilization at a quasi-transparent **m-TiO**_**2**_ electrode. Compared with the immobilization on carbon nanotube in similar conditions^22^, the current density measured in water is lower, but the TiO_2_ inorganic support presents the advantage of being transparent toward a large portion of the sunlight and therefore compatible as a support layer on photoelectrode surfaces.

**Table 1:** (Photo-)electrolysis performances.

System	#	Electrolyte	*t* (h)	*E*_applied_ (V vs. RHE)	$j_{{\mathrm{total}},{\mathrm{TiO}}_{2}}$ (mA cm^−2^)	$\eta_{{\mathrm{CO}}_{2}/{\mathrm{CO}}}$ (mV)	CO		H_2_
							FE [Select.] (%)	TON^*c*^	FE [Select.] (%)
EC	1	0.1 M KHCO_3_ CO_2_ sat. (pH = 6.8)	2	−0.51	1.22	400	63 [80]	1002	16 [20]
	2	0.1 M KH_2_PO_4_/K_2_HPO_4_ Ar sat. (pH = 6.8)	2	−0.51	3.00	NA	0 [0]	NA	86 [100]
PV–EC	3	0.1 M KHCO_3_ CO_2_ sat. (pH = 6.8)	2	0.14	0.90	−250	80 [80]	941	20 [20]
	4	0.1 M KHCO_3_ CO_2_ sat. (pH = 6.8)	2	−0.06	3.25	−50	96 [87]	4060	14 [13]
	5^a^	0.5 M KHCO_3_ CO_2_ sat. (pH = 7.2)	7	−0.03	1.78	−80	NR [82]	NR	NR [18]
PEC	6	0.1 M KHCO_3_ CO_2_ sat. (pH = 6.8)	2	−0.06	0.81	−50	89 [97]	8031	3 [3]
	7^b^	0.1 M KHCO_3_ CO_2_ sat. (pH = 6.8)	2	−0.06	0.05	−50	0 [0]	NA	31 [100]

^a^Under continuous gas flow.

^b^No catalyst.

^c^Lowest estimation (see Methods).

*NA* not applicable, *NR* not reported (experiments under gas flow).

### Hybrid PV–EC reduction of CO_2_

**Fig. 5:**
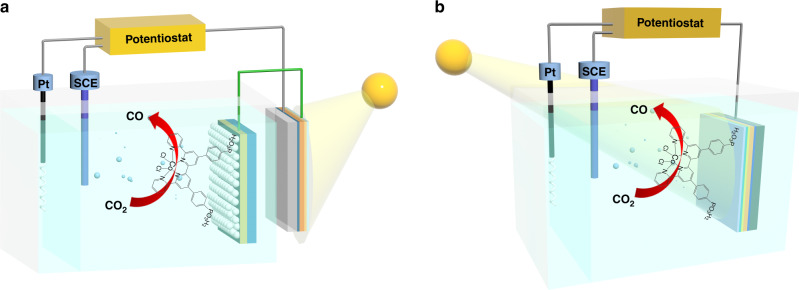
Illustrative schemes of the photo-assisted cell systems investigated in this study. PV–EC system (**a**) and PEC system (**b**).

Considering the good performances of **Co-qPyH**|**m-TiO**_**2**_ electrode in EC conditions, we then used this hybrid material as top layer in association with SCs. Copper chalcogenide SCs, such as p-type CIGS, are appealing materials for the development of CO_2_ reduction photocathodes, but surprisingly they have not been used yet for such application although successful utilization of CIGS for hydrogen evolution has been reported^29–31^. CIGS materials present the advantage to highly absorb in the visible range, their bandgaps are easily tunable through variation of the composition (In/Ga ratio), and they can be manufactured at low cost with current industrial processes^32^. Also note that high-performance and affordable CIGS-based PV cells have led to high power conversion efficiencies (above 23%) while they also have reached the commercial stage^33^. We prepared a p–n junction made of this SC and a n-CdS layer, and the material was further protected by ZnO and ZnO:Al (AZO) layers. Hereafter, CIGS refers specifically to Cu(In_0.1_Ga_0.9_Se_2_) composition and a scanning electron microscopy image was obtained (SEM, Supplementary Fig. 6). *J–V* curve on this CIGS cell (Supplementary Fig. 7) showed a maximum light-to-electricity conversion at 580-mV photovoltage. At this photovoltage, the CIGS cell is delivering ca. 15 mA cm^−2^ current density, compatible with previously reported aqueous EC reduction of CO_2_ to CO catalyzed by Co quaterpyridine complex immobilized on carbon nanotubes (*E* = −0.51 V vs. RHE)^22^. Even though **Co-qPyH**|**m-TiO**_**2**_ electrodes were not yet optimized to reach this range of current densities in EC conditions, we decided to work in this photovoltage range. The working potential under illumination of the cathode was thus set at −0.06 V vs. RHE. Two successive strategies were devised, as illustrated in Figs. 5a, b. First, an external CIGS PV cell (**ZnO:Al**|**ZnO**|**CdS**|**CIGS**|**Mo** material, see “Methods” for details) was coupled with the above **Co-qPyH**|**m-TiO**_**2**_ electrode (Fig. 5a). Second, a fully integrated PEC was set upon depositing **f-TiO**_**2**_ layer directly on top of the **ZnO:Al/ZnO/CdS/CIGS/Mo** stack and further functionalized with **Co-qPyH** (Fig. 5b).

Coupling a PV cell to an electrolyzer (PV + EC) is a valuable approach to utilize the photogenerated charges in the solar cell and activate a substrate in solution. In this configuration, the photoactive material is not in direct contact with the solution, which partly solves stability issues. The **Co-qPyH**|**m-TiO**_**2**_ electrode was connected to CIGS solar cells (**ZnO:Al/ZnO/CdS/CIGS/Mo**), and the photocurrent was measured by recording a linear scan voltammetry (LSV) under chopped light (Supplementary Fig. 8a) showing a photocurrent up to 1 mA cm^−2^. Electrolysis experiments were then carried out in a 0.1 M KHCO_3_ solution saturated with CO_2_ (at pH 6.8) for 2 h. Average current density of 0.90 and 3.25 mA cm^−2^ reported per geometric surface of **m-TiO**_**2**_ was obtained, under applying a bias potential of 0.14 and −0.06 V vs. RHE, respectively (Supplementary Fig. 8b). Analysis of the reaction products indicates that the selectivity toward CO was 87% when the bias potential was −0.06 vs. RHE, which corresponds to a negative overpotential *η* = −50 mV (entries 3 and 4, Table 1). A longer-term, 7-h electrolysis was then performed in a 0.5 M KHCO_3_ electrolyte under continuous flow of CO_2_ (pH 7.2) at a potential of −0.03 V vs. RHE (*η* = −80 mV for CO_2_/CO reaction) (Fig. 4c). The average current density was 1.78 mA cm^−2^ and the selectivity for CO production was maintained between 82 and 85% during the whole course of the experiment (entry 5, Table 1).

### PEC reduction of CO_2_

**Fig. 6:**
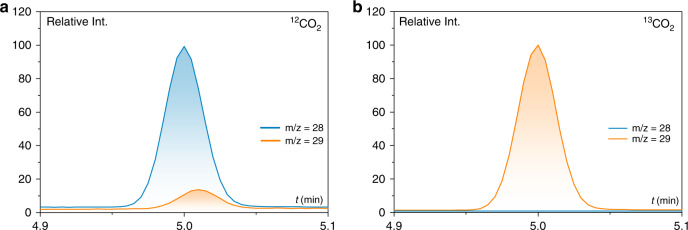
GC/MS chromatograms at the retention time of carbon monoxide. Specific traces of *m*/*z* = 28 value (blue) and *m*/*z* = 29 value (orange) after 2 h of electrolysis at illuminated **Co-qPyH**|**f-TiO**_**2**_|**CIGS** (*E*_bias_ = −0.06 V vs. RHE), in 0.1 M KHCO_3_ electrolyte saturated with ^12^CO_2_ (**a**) and ^13^CO_2_ (**b**).

In a second approach, and as explained above, an integrated PEC with the **Co-qPyH** catalyst directly immobilized on a CIGS-based photocathode was developed (Fig. 5b). To do so, the surface of the complete **ZnO:Al/ZnO/CdS/CIGS/Mo** electrode was first protected by a dense layer of TiO_2_ (5 to 10 nm thick, **f-TiO**_**2**_) deposited by sputtering, and the catalyst was loaded by chemisorption using the same conditions as those used for **m-TiO**_**2**_ film (**Co-qPyH**|**f-TiO**_**2**_|**CIGS**). As expected, a lower catalyst loading was measured by ICP–OES, 3 ± 1 nmol/cm^2^, corresponding to a decrease by a factor 10 as compared with **m-TiO**_**2**_ film. Linear sweep voltammetry with chopped illumination in 0.1 M KHCO_3_ aqueous solution (Fig. 4d) revealed a photoresponse with an onset potential of 0.2 V vs. RHE, while a control experiment under Ar showed negligible photocurrent modulation in the same pH conditions. When long-term photoelectrocatalysis experiment was carried out at a bias potential of −0.06 V vs. RHE (entry 6, Table 1), an average current density of 0.8 mA cm^−2^ was recorded. It showed only a slight decay along the 2 h electrolysis (Supplementary Fig. 9). Selectivity of 97% for CO was obtained with only 3% of H_2_ as the by-product. The decrease in photocurrent density during the photoelectrolysis can be explained by catalyst leaching from the electrode, and/or instability of the composing layers, particularly if the AZO underlayer gets into contact with the aqueous environment. Nonetheless, it is remarkable that such a high selectivity for CO production could be obtained for several hours with this fully integrated device, at a negative overpotential relatively to *E*^0′^(CO_2_/CO). A turnover number for CO production over 8000 was calculated (entry 6, Table 1). It corresponds to a mean turnover frequency (TOF) of 1.1 s^−1^. Note that TON and TOF values are underestimated since they were calculated from the total surface concentration of the catalyst and not from the electroactive fraction of it. A blank experiment was conducted without catalyst grafted on TiO_2_ (entry 7, Table 1) and gave no CO along with low current density. Electrolysis experiment was also repeated with ^13^CO_2_, and analysis of products via GC/MS compared with experiments under ^12^CO_2_ confirmed that CO originated from CO_2_ (Fig. 6).

## Discussion

To the best of our knowledge, this work represents the first demonstration where a CIGS electrode has been successfully used for CO_2_ catalytic reduction. When combined with **Co-qPyH** molecular catalyst grafted on a titanium dioxide upper protective layer, good selectivity, activity, and negative overpotential were simultaneously obtained under visible-light illumination in neutral aqueous solution. It resulted in significant improved performances when compared with previously reported molecular hybrid systems, even those including noble metals (see in particular entries 1, 5, and 6 in Supplementary Table 1). In that latter case, for example, a maximum photocurrent density for CO of 45 μA cm^−2^ at 110 mV overpotential was obtained with a Ru complex grafted onto a hematite photocathode^18^, more than one order of magnitude less than the **Co-qPyH**|**f-TiO**_**2**_|**CIGS** photocathode. Notably, our study led to the first PEC device demonstrating photoelectrocatalytic activity in pure aqueous medium with 97% of selectivity for CO_2_ reduction to CO. It paves the way to the development of better-performing photocathode PECs for CO_2_ reduction. Indeed, the system may be significantly improved with a higher catalyst loading with strategy such as nanostructuration of the upper oxide layer. Eventually, it could also end up in fully unassisted solar-driven fuel production when paired with an appropriate photoanodic reaction.

## Methods

### Catalyst synthesis

**Fig. 7:**
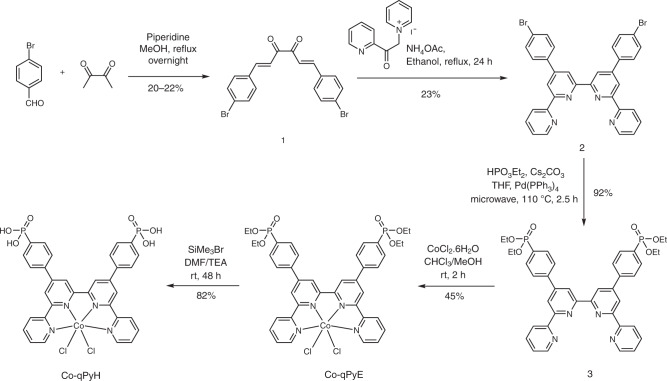
Synthetic route for Co-qPyH. Successive synthetic steps along with chemical yields.

(1E,5E)-1,6-bis(4-bromophenyl)hexa-1,5-diene-3,4-dione (**1**) was synthesized as previously reported in literature and illustrated in Fig. 7^34^. To a vigorously stirred solution of 4-bromobenzaldehyde (11.1 g, 6 mmol) and piperidine (0.6 ml, 0.6 mmol) in MeOH, a solution of 2,3-butadione (2.58 g, 3 mmol) was added dropwise using a dropping funnel over 10 min. The reaction mixture was heated to reflux for overnight. The solution was cooled in an ice bath, and the obtained orange precipitate was filtered and washed with diethyl ether. The orange compound was dried under vacuum to yield 2.71 g of compound **1**. This compound **1** was used directly for the next reaction without any further purification.

4′,4″-bis(4-bromophenyl)-2,2′:6′,2″:6″,2″′-quaterpyridine (**2**): (1E,5E)-1,6-bis(4-bromophenyl)hexa-1,5-diene-3,4-dione (**1**) (420 mg, 1.0 mmol) was added to a solution of the Kröhnke reagent N-[2-(2-pyridyl)-2-oxethyl]pyridinium iodide (652 mg, 3.26 mmol) and anhydrous ammonium acetate (8 g, excess) in absolute ethanol (40 ml) and the mixture heated under reflux for 24 h. After cooling, the precipitate was filtered and washed with diethyl ether. Compound **2** is insoluble in common organic solvents. About 140 mg of compound **2** was obtained as off-white powder with 23% yield. ^1^H NMR characterization is shown in Supplementary Fig. 10 (ppm, 300 MHz, 298 K, CDCl_3_) *δ* 8.88 (d, *J* = 1.8 Hz, 2H), 8.75 (m, 2H), 8.74 (d, *J* = 1.8 Hz, 2H), 8.68 (dt, *J* = 4.8, 1.2 Hz, 2H), 7.92 (td, *J* = 8.1, 1.8 Hz, 2H), 7.79 (d, *J* = 8.7 Hz, 4H), 7.68 (d, *J* = 8.7 Hz, 4H), and 7.38 (dt, *J* = 4.8, 1.2 Hz, 2H); HRMS *m/z* calcd for C_32_H_21_Br_2_N_4_ [M + H]^+^ 619.0133, found 619.0137.

Tetraethyl ([2,2′:6′,2″:6″,2″′-quaterpyridine]−4′,4″-diylbis(4,1-phenylene))bis(phosphonate) (**3**) was formed via Hirao cross-coupling reaction: Compound **2** (100 mg, 0.16 mmol), Pd(PPh_3_)_4_ (17 mg, 0.015 mmol), and Cs_2_CO_3_ (114 mg, 0.35 mmol) were combined in anhydrous THF (4 mL) in a 10- mL microwave vial equipped with a stirrer bar under argon. Diethylphosphite (107 mg, 0.78 mmol) was added by a syringe before the vial and purged with Ar for another 10 min. The vial was sealed and the reaction mixture heated to 110 °C for 2.5 h. The reaction mixture was filtered to give a yellow solution, and then the solvent was evaporated under reduced pressure. The resulting brown residue was dissolved in CH_2_Cl_2_ (20 mL) and stirred with decolorizing charcoal for 30 min and then filtered over celite prior to removal of the solvent under reduced pressure to produce an oily bright-yellow residue. The residue was dissolved in acetone (5 mL) and filtered through a short silica plug eluting with acetone (25 mL) to give a pale-yellow solution. The solvent volume was reduced to ≈5 mL of cold pentane that was added, until compound **3** precipitated out from the solution. Compound **3** was isolated as a white powder (108 mg, 92%). ^1^H NMR characterization is shown in Supplementary Fig. 11 (ppm, 400 MHz, 298 K, CDCl_3_) *δ* 8.95 (d, *J* = 1.6 Hz, 2H), 8.80 (*J* = 1.6 Hz, 2H), 8.75 (m, 2H), 8.70 (dt, *J* = 8.0, 0.8 Hz, 2H), 8.01–8.03 (m, 8H), 7.93 (td, *J* = 7.6, 1.6 Hz, 2H), 7.38 (dt, *J* = 6.0, 1.2 Hz, 2H), 4.13–4.26 (m, 8H), and 1.38 (t, *J* = 6.8 Hz, 12H); ^13^C NMR characterization is shown in Supplementary Fig. 12 (ppm, 100 MHz, 298 K, CDCl_3_) *δ* 156.5, 156.3, 156.1, 149.5, 149.4, 143.0, 137.2, 132.8, 132.7, 127.8, 127.6, 124.3, 121.6, 119.61, 119.56, 62.5, 62.4, 16.6, 16.5; HRMS *m/z* calcd for C_40_H_41_N_4_O_6_P_2_ [M + H]^+^ 735.3501, found 735.3497.

Cobalt[tetraethyl([2,2′:6′,2″:6″,2″′-quaterpyridine]−4′,4″-diylbis(4,1-phenylene)) bis(phosphonate)] bis-chloro (**Co-qPyE**): CoCl_2_.6H_2_O (75.5 mg, 0.31 mmol) was dissolved in methanol (6 mL). A solution of compound **3** (233 mg, 0.31 mmol) in chloroform (6 mL) was added slowly with stirring at ambient temperature. A brown solid was formed gradually upon stirring, and the mixture was stirred for 2 h. The solid was filtered and washed with methanol and chloroform to remove the unreacted ligand and CoCl_2_.6H_2_O. The solid was dried under vacuum to result in yellow powder. Yield: 120 mg (45%). Anal. Calcd. for C_40_H_40_Cl_2_CoN_4_O_6_P_2_: C, 55.57; H, 4.66; N, 6.48. Found: C, 55.70; H, 4.76; N, 6.98; HRMS *m*/*z* calcd for C_40_H_40_CoN_4_O_6_P_2_ [M-2Cl^−^]^2+^ 793.1755, found 793.1762.

Cobalt[2,2′:6′,2″:6″,2″′-quaterpyridine]-4′,4″-diylbis(4,1-phenylene)bis(phosphonic acido)]bis-chloro (**Co-qPyH**): hydrolysis of phosphonate ester groups of **Co-qPyE** could not be accomplished in refluxing at 6 N HCl as usual owing to high risk of decomplexation, so the following procedure was instead followed. **Co-qPyE** (50 mg, 0.06 mmol) was taken in an oven-dried three-neck round-bottom flask kept under Ar atmosphere. About 6 mL of extra dry DMF was added followed by 0.25 mL of distilled triethylamine added to the mixture. Then 0.15 mL of SiMe_3_Br was added to the reaction mixture dropwise, and the reaction mixture was stirred for 48 h at 45 °C. The reaction mixture was cooled, and solvent was removed through reduced pressure. The residue was diluted with very dilute aqueous solution of HCl (pH = 6.8) to remove quaternary ammonium salt formed during reaction and protonation of phosphonic acid. The brown precipitate was filtered and washed with water and dried under vacuum to produce 36 mg (yield = 82%) of Co-qPyH. Anal. Calcd. for C_32_H_24_Cl_2_CoN_4_O_6_P_2_·3.1H_2_O: C, 47.56; H, 3.77; N, 6.93. Found: C, 48.07; H, 4.23; N, 6.93. HRMS *m*/*z* calcd for C_32_H_24_CoN_4_O_6_P_2_ [M-2Cl^−^]^2+^ 681.0503, found 681.0494.

### Nuclear magnetic resonance (NMR)

^1^H and ^13^C NMR spectra were recorded on an *AVANCE 300 UltraShield BRUKER* and *AVANCE 400 BRUKER*. Chemical shifts for ^1^H and ^13^C NMR spectra are referenced relative to residual protium and ^13^C in the deuterated solvent (CDCl_3_ *δ* = 7.26 ppm for ^1^H and *δ* = 77.16 ppm for ^13^C). NMR spectra were recorded at room temperature; chemical shifts are given in ppm and coupling constants in Hz. NMR spectra are available in Supplementary Figs. 10–12.

### HRMS

High-resolution mass (HRMS) spectra were obtained by electrospray ionization coupled with high-resolution ion trap orbitrap (LTQ-Orbitrap, ThermoFisher Scientific) working in ion-positive or ion-negative mode.

### Mesoporous TiO_2_ layer preparation

FTO-conductive glass substrates (F-doped SnO_2_) were purchased from Pilkington (TEC8). Three successive layers of TiO_2_ were then screen-printed using a transparent colloidal paste (Dyesol DSL 18NR-T) with 10-min-long drying steps at 100 °C between each layer. Overall, the thickness of the film was ca. 12 µm. The obtained substrates were then sintered at 450 °C, following a progressive heating ramp (325 °C for 5 min, 375 °C for 5 min, and 450 °C for 30 min).

### Dense thin layer (~5–10-nm thick) of TiO_2_ as protective film deposition

TiO_2_ was RF-sputtered from a ceramic target (3-in. diameter, purity 99.99%) at room temperature. The base pressure in the deposition chamber was lower than 10^−6^ mbar before the sputerring gas (pure Ar) was introduced and the plasma created. Deposition duration was about 20 min with a power of 150 W; these rather soft conditions were applied not to degrade the quality of the CIGS/CdS p–n junction through local heating or any other sputter damages.

### Mott–Schottky measurement

The Mott–Schottky plot was obtained from IMPE-Impedance-Potential measurements using a CHI 760 potentiostat with a **m-TiO**_**2**_|**FTO** electrode (three-electrode configuration). Potential range was 0.5–1.4 V vs. RHE in 1 M KOH (pH 13.9). The frequency was set to 1000 Hz and the amplitude to 0.01 V. Epoxy resin was used to cover any exposed FTO, leaving only the TiO_2_ exposed. The donor density, *N*_*D*_, was calculated from the slopes of the Mott–Schottky plot, using Eq. (1)^35^

1 $$\frac{1}{{C^2}} = \frac{2}{{e\varepsilon \varepsilon _0N_D}}\left( {E - E_{fb} - \frac{{kT}}{e}} \right)$$

with a dielectric constant (ε) of 75 for TiO_2_^36^.

### Adsorption of Co-qPyH on TiO_2_

A 0.2 mM solution of **Co-qPyH** in 10 mL of methanol was prepared; then two drops of water were added, and the mixture was sonicated for 10 min with continuous degassing by Ar. Then electrodes were dipped into this solution, and this bath was heated at 45 °C for 48 h. The electrodes were carefully rinsed with methanol and dried under nitrogen atmosphere.

### UV–vis

Ultraviolet–visible (UV–vis) absorption spectra were recorded with a Variant Cary 300 spectrometer, using 1-cm path-length cells. TiO_2_ surface spectrum shows a slight bathochromic shift of the π–π* intragap transition upon grafting the cobalt quaterpyridine complex **Co-qPyH** (Supplementary Fig. 3) as already observed in the related case^37^.

### ART-IR

ART-IR spectra were recorded on BRUKER Tensor 27.

### Inductively coupled plasma

Cobalt catalyst was desorbed from the electrode with 69% HNO_3_ solution. Solutions were then diluted to 1.38% HNO_3_ and injected into ICP. ICAP 6300 Thermoelectron apparatus was used with detection of cobalt (238.8-nm band that is the most intense).

### X-ray photoelectron spectrometer

X-ray photoelectron spectrometer analyses were performed with a THERMO-VG ESCALAB 250 (RX source K AI (1486.6 eV)).

### CIGS electrode preparation

CuIn_0.1_Ga_0.9_Se_2_ (CIGS) layers were grown onto Mo-coated soda-lime glass substrates by co-evaporation from elemental sources. The soda-lime glass consists of 1-mm-thick microscope slides (1 × 3 in.^2^). Mo back contact was deposited by DC sputtering, with total thickness of 500 nm. The process used for CIGS deposition is known as CuPRO process (for more details see ref. ^38^), which consists of keeping In and Ga fluxes constant and changing Cu flux such that nominal composition of the growing film undergoes Cu-poor/Cu-rich transitions needed for high-performance devices^39^. Substrate temperature was kept constant (580 °C) during the whole deposition, and the thickness of the final films is about 2 µm.

The n-type partner of the junction was a 40-nm-thick CdS grown by chemical bath deposition. The p-CIGS/n-CdS heterojunction was finally covered with RF-sputtered ZnO/ZnO:Al bilayer. For the present study, since two device configurations were used (see below), the applied ZnO:Al-layer thickness was compromised and chosen at 300 nm (*R*_sheet_ = 20 Ω/sq) although usually thinner for such wide-gap absorber-based solar cells. A scanning electron microscope cross-section image of the film is shown in Supplementary Fig. 6.

### *J*(*V*) curve measurement

*J*(*V*) measurements were performed in conditions as close as possible to those defined by standard testing conditions, namely at 25 °C, under AM1.5 (using filtered light source) normalized at 1000 W m^−2^. The size of the investigated solar cells was about 1 × 0.5 cm^2^. The actual value of *J*_sc_ was deduced from the integration of EQE measured from a laboratory-built setup. The surface shaded by the grids, accounted in the *J*_sc_ calculation, is about 3%.

### EC measurements

EC and PEC experiments were performed using a CHI 760 or a PGSTAT 302N Autolab potentiostat–galvanostat. Electrolytes were saturated with the gas of interest by bubbling more than 20 min prior to measurement. A standard three-electrode EC setup was used. Counter electrode (CE) was a platinum grid, separated from the working compartment by a glass frit bridge filled with the same electrolyte. Potentials were measured relative to a saturated calomel electrode (SCE) as reference electrode (Ref) and converted into relative hydrogen electrode (RHE) using Eq. (2)

2 $$E\left( {{\mathrm{V}}{\mathrm{vs.}}{\mathrm{RHE}}} \right) = E\left( {{\mathrm{V}}{\mathrm{vs.}}{\mathrm{SCE}}} \right) + 0.242 + 0.059 \, \times {\mathrm{pH}} .$$

For PV + EC, the external **CIGS** PV cell is connected in series with a **Co-qPyH**|**m-TiO**_**2**_ electrode. Front (−) and back (+) lateral contacts of the cell were taken on ZnO:Al and Mo layers, respectively. When the geometric surface of TiO_2_ is different from the surface of irradiated CIGS, current density is reported per surface of electrode and per surface of PV. When only one single value is reported for current density, it means that both CIGS and TiO_2_ surfaces have been intentionally set to the same value.

In PEC configuration, working electrode was covered with an insulating tape covering the edges to avoid contact between underlayers and electrolyte. A window was previously cut in this tape to define the working area. Working surface was between 0.15 and 0.25 cm^2^.

Irradiation was simulated with a 150-W xenon lamp from Oriel Instrument with light source positioned at 20 cm from the PV cell for PV + EC configuration and 25 cm from the photoelectrode in PEC configuration. UV light was filtered at wavelength below *λ* = 435 nm with a low-pass filter. Part of the light above 1000 nm was also filtered via a water filter to avoid electrolyte warmup. In PEC configuration, the cell was equipped with a quartz window to avoid parasite light absorption.

### Gas chromatography

GC analyses of gas evolved in the headspace during the electrolysis were performed with an Agilent Technologies 7820A GC system equipped with a thermal conductivity detector. Conditions allowed detection of H_2_, O_2_, N_2_, CO, and CO_2_. Calibration curves for H_2_ and CO were determined separately by injecting known volumes of pure gas. From product quantification, TON and TOF are calculated from Eqs. 3 and 4, respectively

3 $${\mathrm{TON}} = n_{{\mathrm{product}}}/n_{{\mathrm{catalyst}},{\mathrm{film}}},$$

4 $${\mathrm{TOF}} = {\mathrm{TON}}/t,$$

Because the electrolysis is not conducted until total deactivation of the catalyst, the given TON value can only be regarded as a lower estimation of the actual value. Also, it is important to recall that in many cases, the amount of electrochemically active molecular catalyst can be an order of magnitude smaller than the amount of catalyst present at the electrode^22,40^. It may also lead to an underestimated value of the calculated TON. Finally, because the amount of active catalyst in the film is decaying over time, the TOF value is an apparent, lowest estimation.

### Chemicals

Chemicals were purchased from Sigma-Aldrich or Alfa Aesar and used as received. Thin-layer chromatography was performed on aluminum sheets precoated with Merck 5735 Kieselgel 60F_254_. Column chromatography was carried out with Merck 5735 Kieselgel 60F (0.040–0.063-mm mesh). All gases were supplied by Air Liquide.

## Supplementary information

Supplementary Information

Peer Review

## Data Availability

Data supporting the findings of this study are available within the article and its Supplementary Information, or from the corresponding authors upon reasonable request.
