# Supplementary material for: Photocathode functionalized with a molecular cobalt catalyst for selective carbon dioxide reduction in water
Source: Nat Commun. 2020 Jul 13;11:3499. doi: 10.1038/s41467-020-17125-4 (PMC7358214; doi:10.1038/s41467-020-17125-4)
Supplement: Supplementary file 1 — Supplementary Information [file 41467_2020_17125_MOESM1_ESM.pdf]

Supplementary Information

Photocathode functionalized with a molecular cobalt catalyst for selective carbon dioxide reduction in water

Pati *et al.*

Supplementary table 1. Summary of the most efficient molecular photocathodes for CO_2 reduction reported in the literature.

Entry	Light absorbing electrode	Catalyst	$E_{applied}$	J_{total} (mA cm^{-2}) @ $E_{applied}$	J_{CO} (mA cm^{-2}) @ $E_{applied}$	FE total (%) @$E_{applied}$	FE_{CO} (%)@ $E_{applied}$	Selectivity for CO@ $E_{applied}$	Electrolyte	Illumination	Ref.
1	Fe_2O_3 co-doped with N and Zn| TiO_2	Ru(MeCN)CO_2C 3Py-p	0.1V vs.RHE	0.15	0.045	100<br>13h	30<br>13h	30<br>13h	0.1 M $KHCO_3$(aq.)	100 mW cm^{-2}, AM1.5G	1
2	Cu_2O | TiO_2	ReCl(bpy)$(CO)_3$	-2.05 V *vs.* Fc^+/Fc	2.5 *<br>@-2.0 V<br>*vs.*Fc^+/Fc	≈2~2.4<br>@-2.0 V<br>*vs.*Fc^+/Fc	NR	NR	80~95<br>1.5h	0.1 M $TBAPF_6$, MeCN	100 mW cm^{-2}, AM1.5G	2
3	SiNWs−H	MnBr(bpy)$(CO)_3$	-1.10 V vs. SCE	≈1.00 *<br>@-1.2 V *vs.* SCE	≈1.00<br>@-1.2 V *vs.* SCE	NR	NR	100<br>3h	CH_3CN + 0.1 M Bu_4NClO_4 under CO_2 + 5% v/v H_2O	Hg-Xe lamp, 525 < λ < 655 nm	3
4	NiO | Zn-porphyrin	ReCl(bpy)$(CO)_3$	NR	0.020 (mA)	NR	NR	6.2<br>115h	NR	0.1 M $TBAPF_6$, DMF	Xe lamp, λ = 430 nm	4
5	NiO	Ru(II)−Re(I) dyad	-0.11 V vs.RHE	≈ 0.005	≈0.0045	64%<br>12h	58	91<br>12h	0.05 M $NaHCO_3$(aq.)	Xe lamp, λ = 460 nm	5
6	$CuGaO_2$	Ru(II)–Re(I) dyad	0.29 V vs.RHE	0.011 *<br>0.0 V vs.RHE	≈0.007<br>0.0 V vs.RHE	81<br>15h	49<br>15h	61<br>15h	0.05 M $NaHCO_3$(aq.)	Xe lamp, λ = 460 nm	6
7	Si | TiO_2	Co(II) bis-terpyridine	0.0 V vs.RHE	0.18	0.029	63<br>8h	9.5<br>8h	15<br>8h	0.1 M $KHCO_3$(aq.)	100 mW cm^{-2}, AM1.5G	7
			−1.0 V vs. Fc+/0	0.25	0.13	77<br>24h	41<br>24h	53<br>24h	0.1 M $TBABF_4$, 6:4(v:v) MeCN:H_2O		
8	Si | n-GaN	RuCt	-0.25 V vs. RHE	≈1.1	≈0.7	69<br>20h	64 for formate<br>20h	93 for formate<br>20h	0.05 M $NaHCO_3$(aq.)	100 mW cm^{-2}, AM1.5G	8
PV-EC system	CIGS	Co(II) quaterpyridine CoqPyH	-0.06 V vs.RHE	0.9	0.78	110<br>2h	96<br>2h	87<br>2h	0.1 M $KHCO_3$(aq.)	Xe lamp, λ > 435 nm	This work
			-0.033 V vs.RHE	1.78	1.50	NR	NR	82<br>7h	0.5 M $KHCO_3$(aq.)		
PEC system			-0.06 V vs.RHE	0.81	0.72	92<br>2h	89<br>2h	97<br>2h	0.1 M $KHCO_3$(aq.)		

* Estimated from LSV measurements; NR = not reported.

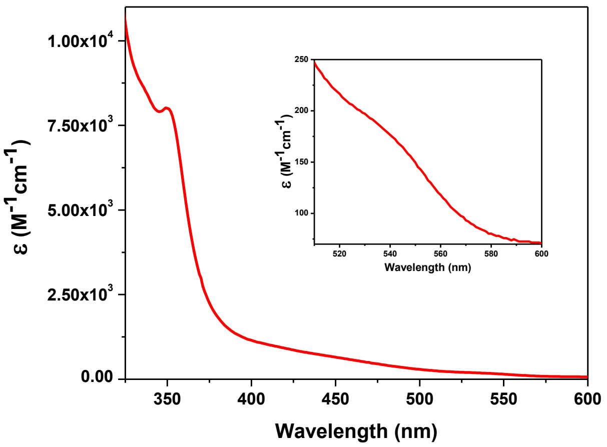

Supplementary Figure 1. Electronic absorption spectrum of compound **Co-qPyE** recorded in DMF. **Co-qPyH** is partly soluble in MeOH solution, whereas **Co-qPyE** is fairly soluble in acetonitrile and DMF. The electronic absorption spectra of **Co-qPyE** exhibits a strong absorption band at 350 nm, which is assigned to the intra ligand π-π* transition on the quaterpyridine. The weak and broad band with maxima at 448 nm and 540 nm can be attributed to the metal-to-ligand charge transfer (MLCT) transition for **Co-qPyH**.

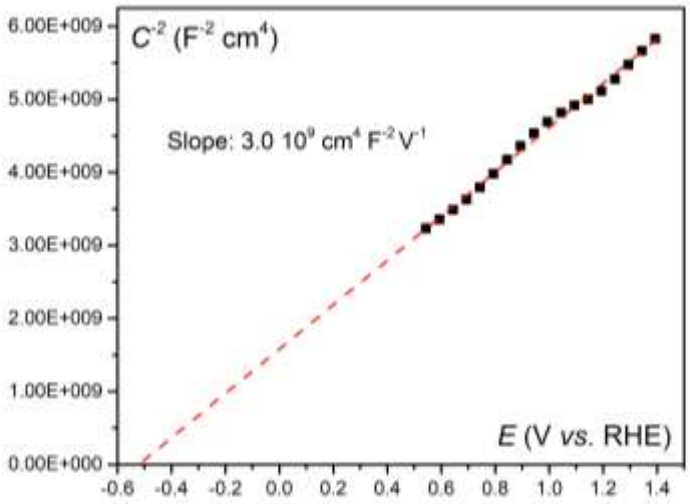

Supplementary Figure 2. Mott-Schottky plot in 1 M KOH solution for a TiO_2 film deposited onto FTO.

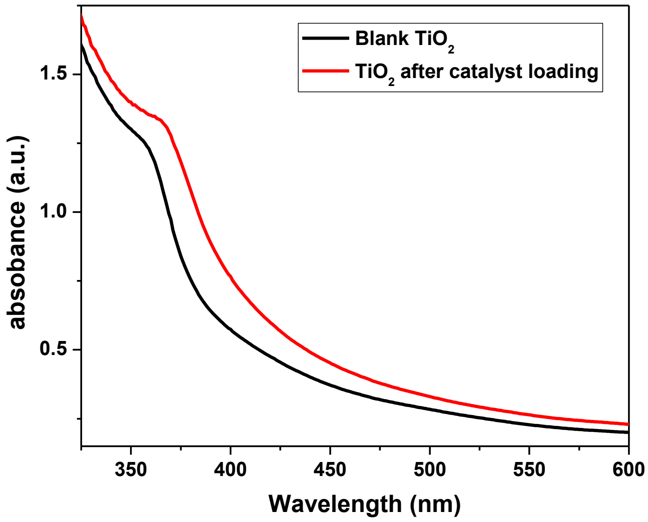

Supplementary Figure 3. UV-Vis absorption spectrum of **Co-qPyH** grafted on **m-TiO_2** film.

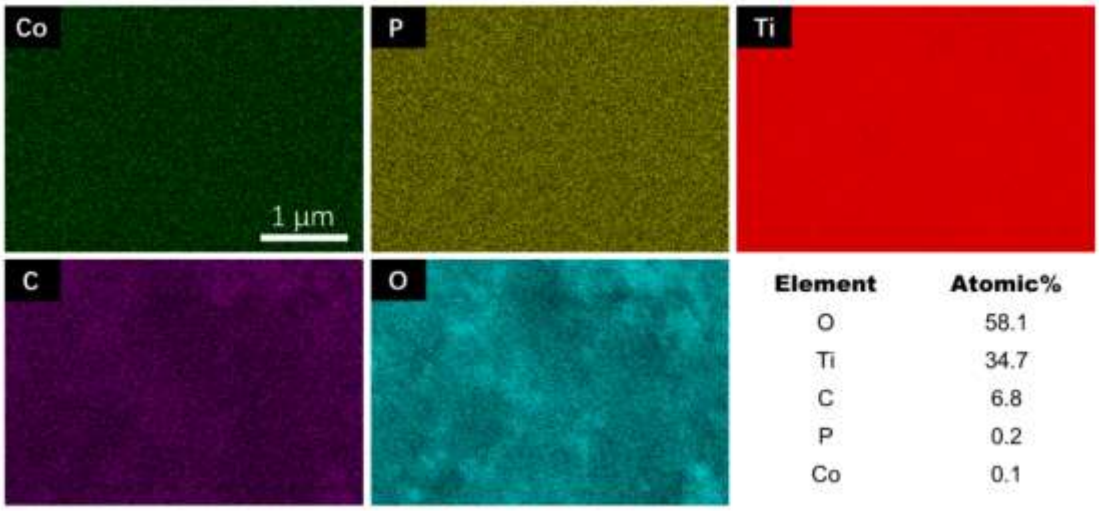

Element	Atomic%
O	58.1
Ti	34.7
C	6.8
P	0.2
Co	0.1

Supplementary Figure 4. EDX-mapping images of a **Co-qPyH|m-TiO_2** electrode.

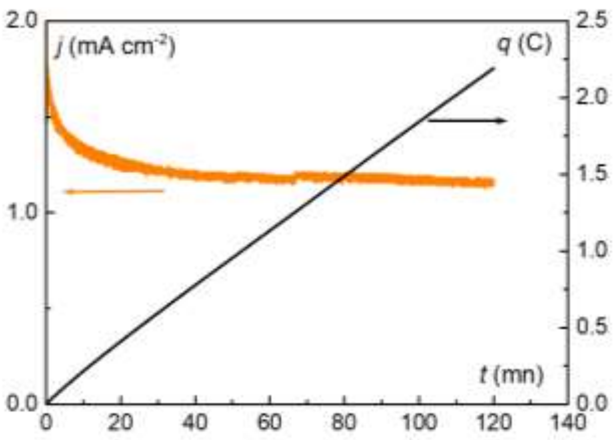

Supplementary Figure 5. Long-term electrolysis in EC conditions at **Co-qPyH|m-TiO_2** polarized at -0.51 V *vs.* RHE in 0.1 M $KHCO_3$ electrolyte saturated with CO_2 (pH 6.8).

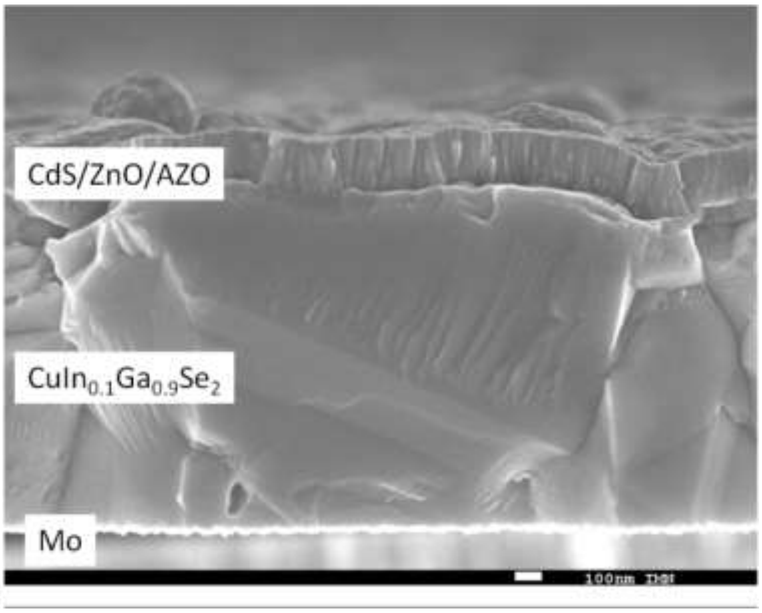

Supplementary Figure 6. Scanning electron microscope (SEM) cross section image of the CIGS layer.

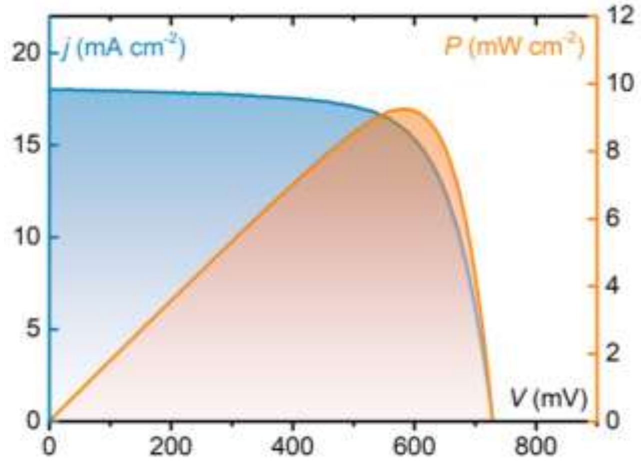

Supplementary Figure 7. *J-V* curve of one of the CIGS solar cell without TiO_2 layer (front contact made with a grid directly on AZO). The CIGS based solar cell exhibits a short-circuit photocurrent density (J_{sc}) of 18 mA cm^{-2} (calculated from the external quantum efficiency, EQE), and an open circuit voltage (V_{OC}) ranging between 700 and 750 mV from one cell to another. The fill factor (*FF*) ranges between 60 and 70 % resulting in a power conversion efficiency of about 9 % under AM 1.5 (1000 W/m^2).

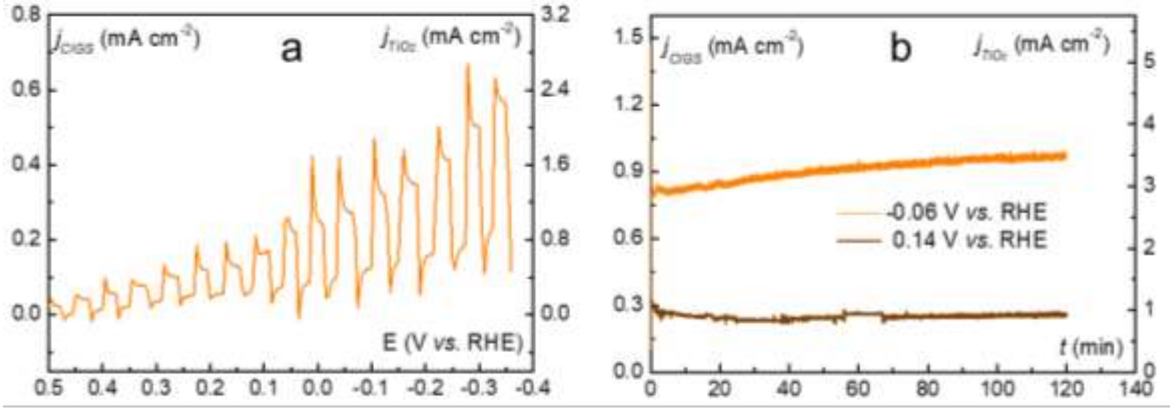

Supplementary Figure 8. (**a**) LSV in PV+EC conditions at **Co-qPyH|m-TiO2** electrode in a 0.1 M $KHCO_3$ electrolyte saturated with CO_2 (pH 6.8) and connected to an external CIGS solar cell under chopped light illumination. Scan rate was 5 mV s^{-1}. (**b**) Electrolysis in PV+EC conditions at **Co-qPyH|m-TiO2** electrode in a 0.1 M $KHCO_3$ electrolyte saturated with CO_2 (pH 6.8), and connected to an external CIGS solar cell under light illumination, polarized at +0.14 (brown) and -0.06 (orange) V *vs.* RHE.

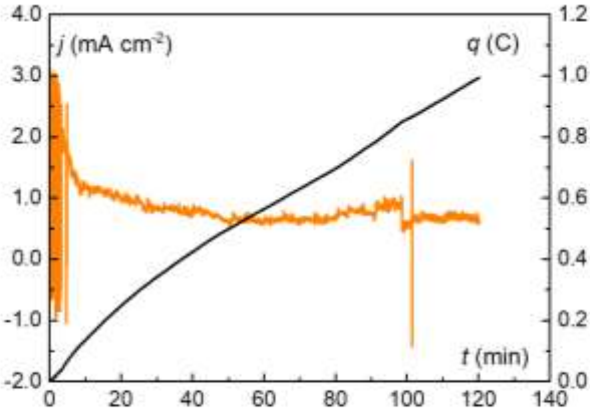

Supplementary Figure 9. Electrolysis in PEC conditions at **Co-qPyH|f-TiO$_2$|CIGS** electrode in a 0.1 M $KHCO_3$ electrolyte saturated with CO_2 (pH 6.8), under light illumination, polarized at -0.06 V *vs.* RHE.

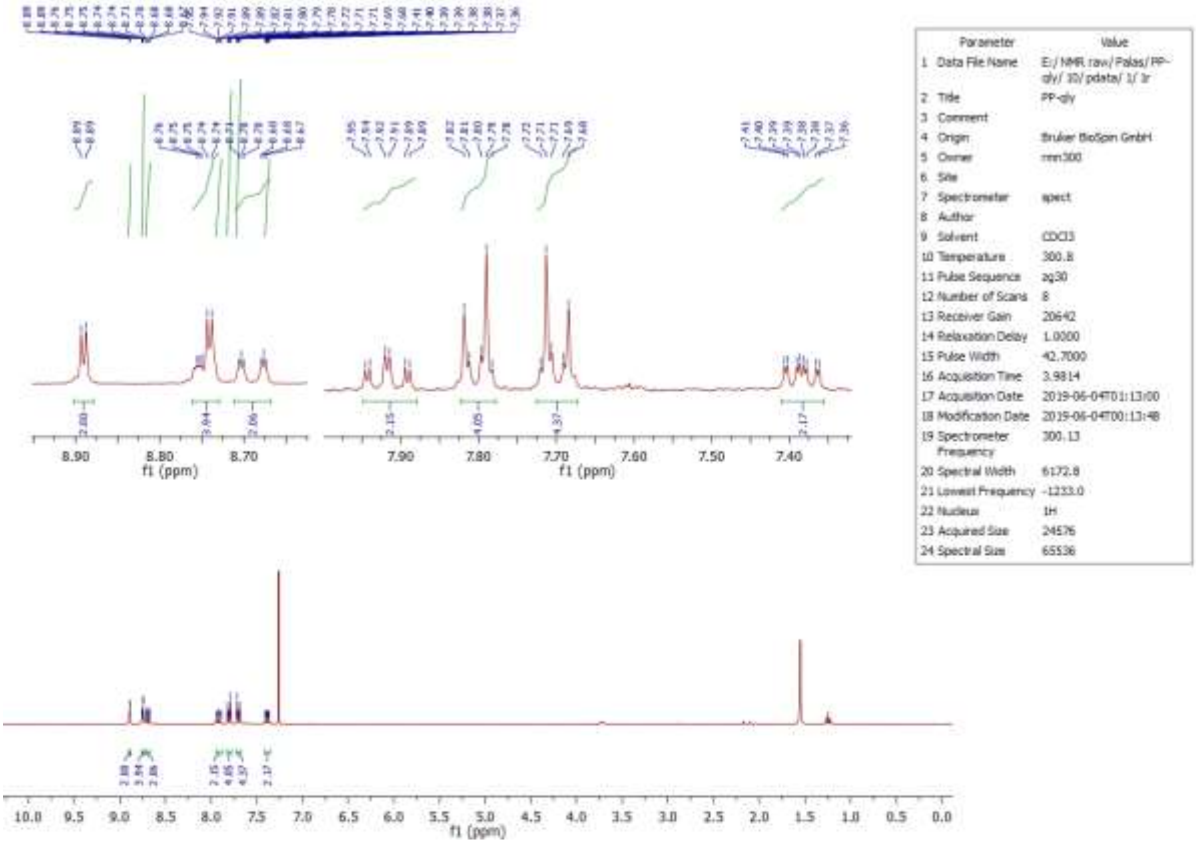

Supplementary Figure 10. ^{1}H NMR spectrum of compound **2** in $CDCl_3$.

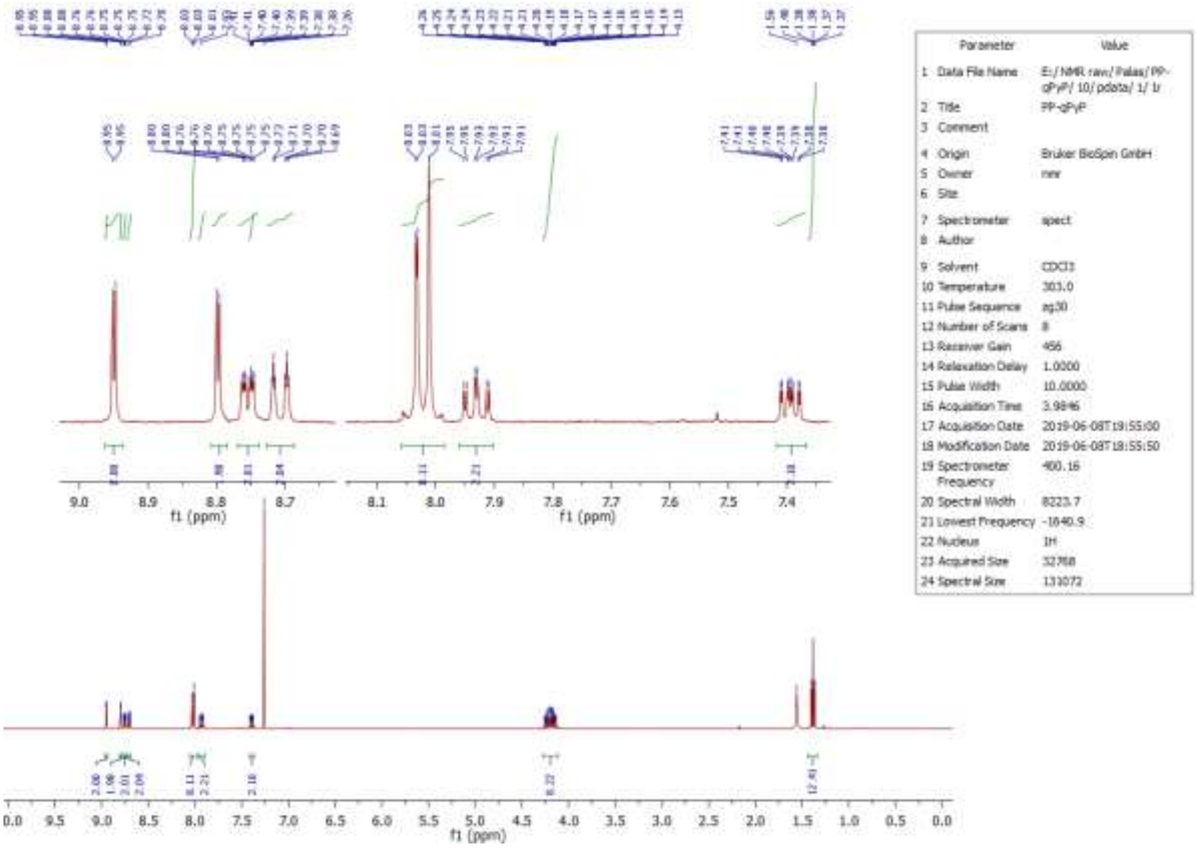

Supplementary Figure 11. ^{1}H NMR spectrum of compound **3** in $CDCl_3$.

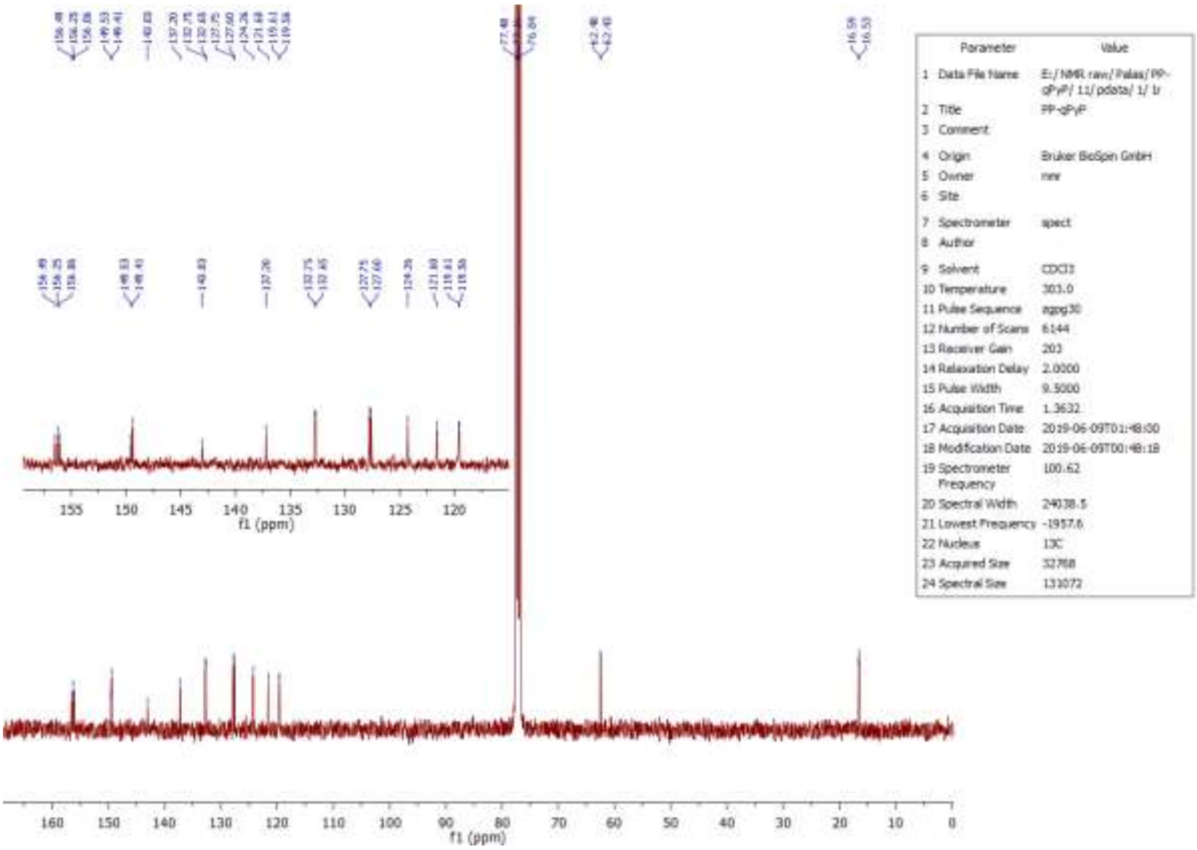

Supplementary Figure 12. ^{13}C NMR spectrum of compound **Co-qPyE** in $CDCl_3$.

Supplementary References

1 Sekizawa, K., Sato, S., Arai, T. & Morikawa, T. Solar-Driven Photocatalytic CO_2 Reduction in Water Utilizing a Ruthenium Complex Catalyst on p-Type Fe_2O_3 with a Multiheterojunction. *ACS Catal.* **8**, 1405-1416, (2018).

2 Schreier, M. *et al.* Covalent Immobilization of a Molecular Catalyst on Cu_2O Photocathodes for CO_2 Reduction. *J. Am. Chem. Soc.* **138**, 1938-1946, (2016).

3 Torralba-Peñalver, E., Luo, Y., Compain, J.-D., Chardon-Noblat, S. & Fabre, B. Selective Catalytic Electroreduction of CO_2 at Silicon Nanowires (SiNWs) Photocathodes Using Non-Noble Metal-Based Manganese Carbonyl Bipyridyl Molecular Catalysts in Solution and Grafted onto SiNWs. *ACS Catal.* **5**, 6138-6147, (2015).

4 Kou, Y. *et al.* Visible light-induced reduction of carbon dioxide sensitized by a porphyrin–rhenium dyad metal complex on p-type semiconducting NiO as the reduction terminal end of an artificial photosynthetic system. *J. Catal.* **310**, 57-66, (2014).

5 Sahara, G. *et al.* Photoelectrochemical Reduction of CO_2 Coupled to Water Oxidation Using a Photocathode with a Ru(II)–Re(I) Complex Photocatalyst and a CoO_x/TaON Photoanode. *J. Am. Chem. Soc.* **138**, 14152-14158, (2016).

6 Kumagai, H. *et al.* Hybrid photocathode consisting of a $CuGaO_2$ p-type semiconductor and a Ru(II)–Re(I) supramolecular photocatalyst: non-biased visible-light-driven CO_2 reduction with water oxidation. *Chem. Sci.* **8**, 4242-4249, (2017).

7 Leung, J. J. *et al.* Solar-driven reduction of aqueous CO_2 with a cobalt bis(terpyridine)-based photocathode. *Nat. Catal.* **2**, 354-365, (2019).

8 Shan, B. *et al.* Binary molecular-semiconductor p–n junctions for photoelectrocatalytic CO_2 reduction. *Nat. Energy* **4**, 290-299, (2019).
