# Supplementary material for: Photocathode functionalized with a molecular cobalt catalyst for selective carbon dioxide reduction in water
Source: Nat Commun. 2020 Jul 13;11:3499. doi: 10.1038/s41467-020-17125-4 (PMC7358214; doi:10.1038/s41467-020-17125-4)
Supplement: Supplementary file 2 — Peer Review [file 41467_2020_17125_MOESM2_ESM.pdf]

Reviewer #1 (Remarks to the Author):

Pati et al. reported a photocathode based on Cu(In,Ga)Se2 (CIGS) SC and cobalt quaterpyridine catalyst for the selective photoreduction of CO2 into CO with a maximum current density of 3.35 mA cm-2 at a bias potential of -0.15 V vs. RHE, which is an impressive performance with novelty. However, some explanation/experiments are confusing. Balancing all these considerations, I can not recommend its publication at this stage, but would be a reasonable candidate after addressing bellow concerns:

(1) In Fig.S6, after catalyst grafting upon TiO2 surface, the UV-Vis absorption spectrum has changed significantly. Therefore, the factors to influence the spectrum variety should be discussed in detail.

(2) In Fig.S3, a N1s characteristic signal at 399.5 eV is not pyridinic nitrogen, but metal-N (Mater. Res. Bull. 2012, 47, 2188-2192). Moreover, why P2p analysis is 1000 counts but not 2000 counts.

(3) In Fig.3, before and after 2 h electrolysis at -0.51 V vs. RHE, the difference of Co2p spectrum is relatively large, please explain it.

(4) In Fig. 4, I wonder why the current density decreases sharply during 2 h electrolysis.

Reviewer #2 (Remarks to the Author):

The authors report the coupling of a cobalt quaternarypyridine catalyst (Co-qPyH) to a Cu(In,Ga)Se2 photocathode to generate a system that shows high activity and selectivity for photoelectrochemical CO2 reduction in aqueous neutral carbonate buffer. Although the Co quaternarypyridine catalysts have been extensively studied by this group for electrochemical and photochemical CO2 reduction, the combination with a CIGS photocathode is a novel and impactful advance. The photoelectrochemical system reduces CO2 to CO at -0.15 V under illumination with a current density for CO of 0.72 mA cm⁻² and a TOF of ~1.1 s⁻¹ over a 2 h experiment.

This is a well-considered study that reports an important advance in the use of this class of Co complexes for CO2RR in aqueous solution, and I believe it has sufficient novelty and impact for publication in Nature Communications. I have only a few very minor suggested changes the authors should consider before publication:

1)In the abstract, the authors state: “Yet, there is no catalytic system able to reduce selectively CO2 into CO in aqueous media with high activity, and using earth-abundant molecular catalyst.” This is not true. There are several recent examples of surface-immobilized molecular catalysts that reduce CO2 to CO in water: Re(tBu-bpy) (Kubiak), CoPc (Wang, McCrory, Manthiram), CoTPPS (Sakai), Ni(cyclam) (Kubiak, Shafaat, Ren, Fujita, etc.), among other. This statement should be removed or clarified.

2)The author should consider reporting TOF (per second) in addition to TON for their measurements in Table 1 and comparisons in Table 2. This might help readers make broader comparisons to the electrocatalysis and photoelectrocatalysis literature.

Reviewer #3 (Remarks to the Author):

This manuscript by Robert, Odobel, Barreau and collaborators reports on the immobilization of a cobalt polypyridyl CO2 reduction catalyst and its integration on a photocathode based on Cu(In,Ga)Se2 semiconductor. The known cobalt quaterpyridine electrocatalyst is interfaced with a

know light harvesting system to obtain a high performing engineered system for CO2 reduction to CO. The key points emphasized by the authors are that this photocathode does not contain noble metals, and has "a good selectively for CO or formate, a near zero overpotential and large current densities when working in aqueous solution." Further claims made by the authors are that this work "represents the first demonstration where a CIGS electrode has been successfully used for CO2 catalytic reduction" and is "the first PEC device demonstrating photoelectrocatalytic activity in pure aqueous medium with 99% of selectivity for CO2 reduction to CO"
While every component of the assembly is known, the photoelectrochemical results obtained are convincing and represent a leap in performance compared to previous systems.

Comments:
• In the abstract the authors claim that their study uses "Atmospheric CO2 dissolved in aqueous solution". Most people would take that to mean CO2 at atmospheric concentrations. This is clearly not the case in the paper, and language in the abstract should be edited to avoid confusion.
• The XPS data in figure 3 shows that most of the Co signals are lost on the surface after electrolysis. Could the authors calculate how much was lost? The electrolysis current traces do not appear to show enough current loss to account for the loss of Co observed. What is the fate of the cobalt?
• Similarly, they authors mention that "Complementary EDX mapping experiment (Fig. S7) shows a consistent atomic ratio of 2 to 1 between phosphate and cobalt, definitively proving the presence of the catalyst and confirming its intactness after grafting." Performing this analysis again after electrocatalysis would be necessary to demonstrate that the cobalt complex stays intact on the surface during CO2 reduction and is the active catalyst.
• ICP-OES (data not shown?) was used to determine catalyst loading on the electrode. The authors write "catalyst loading of 29 ± 4 nmol cm-2 on m-TiO2 film and 3 ± 1 nmol cm-2 in f-TiO2 film (see Methods for details). This concentration is about one third of previously reported data (85 nmol cm-2 for a Re complex with a similar anchoring group on mesoporous TiO2),17 leaving room for improvement." The catalyst loading is used to determine the turn-over-numbers reported after 2h. Given the small numbers involved, any error in the ICP-OES would have a huge impact on the reported performance of the catalyst. Coupled to the fact that the authors are surprised by the low catalyst loading, the experiment should be repeated to get an understanding of the potential error bars on the measurement.
• The meaning of "TON" should be clarified in Table 1: it currently implies that the catalyst is completely degraded during the experiment. Given the current density traces, this does not appear to be the case. Clarifying the definition used or reporting the TOF during the 2-hour of the experiment would be less confusing.
• Figure 6: why did the retention time change for 13CO2 when shifting from natural abundance to 13C labeled CO2? What was changed in the method?
• Why were the electrolyte concentration, solution pH, and overpotential all changed for the 7h electrolysis (table 1 entry 5)? This makes for a difficult comparison to the rest of the work presented.

Reviewer #1 (Remarks to the Author):
Pati et al. reported a photocathode based on Cu(In,Ga)Se2 (CIGS) SC and cobalt quaterpyridine catalyst for the selective photoreduction of CO2 into CO with a maximum current density of 3.35 mA cm-2 at a bias potential of -0.15 V vs. RHE, which is an impressive performance with novelty. However, some explanation/experiments are confusing. Balancing all these considerations, I can not recommend its publication at this stage, but would be a reasonable candidate after addressing bellow concerns:
(1). In Fig.S6, after catalyst grafting upon TiO2 surface, the UV-Vis absorption spectrum has changed significantly. Therefore, the factors to influence the spectrum variety should be discussed in detail.
It is common that the grafting of a molecule (e.g. a chromophore) on TiO_2 surface induces some changes of its absorption spectrum as it is generally observed in the field of dye-sensitized solar cell (see for example: *J. Mater. Chem. A*, 2017, 5, 19541–19559). In the specific case of **Co-qPyH**, we can essentially observe a slight bathochromic shift of the π-π* transition. These changes can be attributed to: i) a modification of the environment around the complex from solution to air, which has a different permittivity; ii) the formation of aggregates on the surface due to the packing, which causes excitonic coupling between the molecules and lastly iii) the modification of the electronic properties of the complex upon chemisorption coming from the anchoring group binding. A comment was added in the ESI below Figure S6 to clarify this point. Thanks for pointing out the issue.

(2) In Fig.S3, a N1s characteristic signal at 399.5 eV is not pyridinic nitrogen, but metal-N (Mater. Res. Bull. 2012, 47, 2188-2192). Moreover, why P2p analysis is 1000 counts but not 2000 counts.
Considering the question, we understand it is related to Fig. 3 (XPS measurements) and not Fig. S3.
-The point here is semantic in fact: what the reviewer calls metal-N is exactly what we call pyridinic nitrogen, as the catalyst features 4 pyridinic nitrogen atoms complexing the cobalt. We thus rephrased the sentence to make it clearer (page 5, revised MS). We have also added a reference to a recent paper describing a cobalt quaterpyridine complex immobilized on carbon nanotubes (see Ref. 22 in the revised MS). In this article, the nitrogen peak falls at 399.4 eV.
-Regarding the P2p scale, it was simply adapted to feature the best zoom possible on the peak, so that the 1000 counts is correct.
Again we thank the reviewer and hope having clarifying these points.

(3) In Fig.3, before and after 2 h electrolysis at -0.51 V vs. RHE, the difference of Co2p spectrum is relatively large, please explain it.
Two explanations can be given for the difference. Regarding intensity decrease, we attribute it to partial desorption of the catalyst. This point is already mentioned in the main text. Of course the loss of intensity makes the peak of cobalt after electrolysis harder to interpret. Importantly, the pattern is conserved and slightly shifted toward lower energy. Since the satellite peak at 786 eV is conserved, we know it is Co(II). The shift of the cobalt signal can be explained by the fact that Co is coordinated to chloride ion before electrolysis, a withdrawing ligand. After electrolysis we don't know what is the coordinating axial ligand to Co, but it cannot be chloride since electrolyte is $KHCO_3$. The presence of a less withdrawing ligand (e.g. water) will account for the observed shift.

(4) In Fig. 4, I wonder why the current density decreases sharply during 2 h electrolysis.
It is actually an interesting point for which we don't have final answer yet. This decrease could be an effect of catalyst desorption that we already discussed in the text. But we prefer not to give more detailed interpretation at this stage.

General comment: there was a typo in Table 1 and in text, selectivity for CO in PEC conditions is 97% and not 99%, we apologize to the reviewer. It has been corrected.

Reviewer #2 (Remarks to the Author):
The authors report the coupling of a cobalt quaternarypyridine catalyst (Co-qPyH) to a Cu(In,Ga)Se2 photocathode to generate a system that shows high activity and selectivity for photoelectrochemical CO2 reduction in aqueous neutral carbonate buffer. Although the Co quaternarypyridine catalysts have been extensively studied by this group for electrochemical and photochemical CO2 reduction, the combination with a CIGS photocathode is a novel and impactful advance. The photoelectrochemical system reduces CO2 to CO at -0.15 V under illumination with a current density for CO of 0.72 mA cm^{-2} and a TOF of ~1.1 s^{-1} over a 2 h experiment.
This is a well-considered study that reports an important advance in the use of this class of Co complexes for CO2RR in aqueous solution, and I believe it has sufficient novelty and impact for publication in Nature Communications. I have only a few very minor suggested changes the authors should consider before publication:

1)In the abstract, the authors state: “Yet, there is no catalytic system able to reduce selectively CO2 into CO in aqueous media with high activity, and using earth-abundant molecular catalyst.” This is not true. There are several recent examples of surface-immobilized molecular catalysts that reduce CO2 to CO in water: Re(tBu-bpy) (Kubiak), CoPc (Wang, McCrory, Manthiram), CoTPPS (Sakai), Ni(cyclam) (Kubiak, Shafaat, Ren, Fujita, etc.), among other. This statement should be removed or clarified.
Thanks for the suggestion. There have been indeed many molecular supported catalysts recently used for the electrochemical CO_2 reduction in water, and we have participated actively to these efforts. In the highlighted sentence, we narrow down the scope to photoelectrochemical conditions, so that our statement is only related to this sub-field. But we agree it can lead to misunderstanding so we have modified the sentence in the introduction to make it clearer.

2)The author should consider reporting TOF (per second) in addition to TON for their measurements in Table 1 and comparisons in Table 2. This might help readers make broader comparisons to the electrocatalysis and photoelectrocatalysis literature.
We calculated the TOF for our data and gave the most pertinent value through the text (we now give this TOF for PEC cell configuration). However, these data should be used with care for comparison since we used for calculation, like in many contributions, the total amount of catalyst on the electrode and not the electrochemically active amount of catalyst (meaning that the TON and TOF values we are giving are underestimated values); we added a footnote to Table 1 and a sentence on page 10 of the MS.
Note also that electrolysis are usually not performed in kinetic conditions (instead there is often diffusion limitation), so that an average catalyst on a gas diffusion electrode may have a better TOF than a really good one in H-cell configuration…
We inserted a comment on Methods to mention these facts and alert the readers.

General comment: there was a typo in Table 1 and in text, selectivity for CO in PEC conditions is 97% and not 99%, we apologize to the reviewer. It has been corrected.

Reviewer #3 (Remarks to the Author):
This manuscript by Robert, Odobel, Barreau and collaborators reports on the immobilization of a cobalt polypyridyl CO2 reduction catalyst and its integration on a photocathode based on Cu(In,Ga)Se2 semiconductor. The known cobalt quaterpyridine electrocatalyst is interfaced with a known light harvesting system to obtain a high performing engineered system for CO2 reduction to CO.
The key points emphasized by the authors are that this photocathode does not contain noble metals, and has "a good selectively for CO or formate, a near zero overpotential and large current densities when working in aqueous solution." Further claims made by the authors are that this work "represents the first demonstration where a CIGS electrode has been successfully used for CO2 catalytic reduction" and is "the first PEC device demonstrating photoelectrocatalytic activity in pure aqueous medium with 99% of selectivity for CO2 reduction to CO".
While every component of the assembly is known, the photoelectrochemical results obtained are convincing and represent a leap in performance compared to previous systems.

Comments:
• In the abstract the authors claim that their study uses "Atmospheric CO2 dissolved in aqueous solution". Most people would take that to mean CO2 at atmospheric concentrations. This is clearly not the case in the paper, and language in the abstract should be edited to avoid confusion.
We agree with the comment, thanks for bringing this to our attention. The text has been edited.

• The XPS data in figure 3 shows that most of the Co signals are lost on the surface after electrolysis. Could the authors calculate how much was lost? The electrolysis current traces do not appear to show enough current loss to account for the loss of Co observed. What is the fate of the cobalt?
We calculated the loss to be ca. 69% from the XPS data.
Generally speaking, considering molecular catalysts on electroactive surface, only a fraction is usually active. Sometimes it could be even less than 10%. It may thus lead to inaccurate interpretation (we are actually preparing a dedicated paper on this topic). If the current is proportional to the amount of catalyst (which is not always the case), this proportionality is related to the amount of active catalyst and not to the amount of total catalyst (while XPS probes the total amount). In any case, the fact that some of the catalyst is desorbing from the electrode is evident and we clearly highlight this in our main text. We added a paragraph on Method to inform the authors on this aspect.

• Similarly, the authors mention that "Complementary EDX mapping experiment (Fig. S7) shows a consistent atomic ratio of 2 to 1 between phosphate and cobalt, definitively proving the presence of the catalyst and confirming its intactness after grafting." Performing this analysis again after electrocatalysis would be necessary to demonstrate that the cobalt complex stays intact on the surface during CO2 reduction and is the active catalyst.
The catalyst intactness has been evidenced by XPS and IR analysis. Additionally, the selectivity does not change until the end of the electrolysis, further supporting catalyst stability. What we know is that there is detachment of a fraction of the catalyst from the electrode; we mentioned it in the paper. As indicated by the reviewer, the EDX mapping is complementary but it is not informative on the catalyst structure as it is simply providing an atomic ratio. We do not think this data would add additional support.

• ICP-OES (data not shown?) was used to determine catalyst loading on the electrode. The authors write "catalyst loading of 29 ± 4 nmol cm-2 on m-TiO2 film and 3 ± 1 nmol cm-2 in f-TiO2 film (see Methods for details). This concentration is about one third of previously reported data (85 nmol cm-2

for a Re complex with a similar anchoring group on mesoporous TiO2),17 leaving room for improvement.” The catalyst loading is used to determine the turn-over-numbers reported after 2h. Given the small numbers involved, any error in the ICP-OES would have a huge impact on the reported performance of the catalyst. Coupled to the fact that the authors are surprised by the low catalyst loading, the experiment should be repeated to get an understanding of the potential error bars on the measurement.

The ICP measurement was done twice for each measurement with an error below 1%. What makes the error larger is the amount of catalyst deposited through the adsorption step. But as said above, the amount of active catalyst is usually way smaller than the amount of deposited catalyst so that these TON values should be regarded as lowest estimation of the TON rather than accurate TON value. This is why we did not to give error bar on the TON. We added a paragraph in methods to alert the reader on this fact.

• The meaning of “TON” should be clarified in Table 1: it currently implies that the catalyst is completely degraded during the experiment. Given the current density traces, this does not appear to be the case. Clarifying the definition used or reporting the TOF during the 2-hour of the experiment would be less confusing.

Thanks for raising this point. We agree that TON is usually not used properly and made specific paragraph on the method part to answer both this point and the one above. Note that our values are underestimated as explained in the method section.

• Figure 6: why did the retention time change for 13CO2 when shifting from natural abundance to 13C labeled CO2? What was changed in the method?

The small discrepancy is issued from manual injection in GC/MS apparatus. Manual injection with a syringe implies manual triggering for recording the spectra. As a consequence, there could be a small variation in the retention time. In the present case, $^{13}CO_2$ is shifted by less than 0.01 minutes, which represents only 0.5 second.

• Why were the electrolyte concentration, solution pH, and overpotential all changed for the 7h electrolysis (table 1 entry 5)? This makes for a difficult comparison to the rest of the work presented.

Actually, only the electrolyte concentration is slightly changed in this experiment, and the small differences in pH and overpotential values are due to this larger concentration. The aim of this electrolysis was not to give a comparison point, but to show that the durability of the system could be extended beyond 2h.

As far as comparison is concerned, various set-up are comparable for 2h electrolysis time, with 0.1 M $KHCO_3$ electrolyte. PV-EC experiment can indeed be compared with PEC configuration upon considering data from entries 4 and 6 respectively (Table 1), obtained at the same overpotential (-50 mV). Comparison with pure electrolysis conditions can be made with entry 1 data (which were obtained at large overpotential (400 mV), that is the cost for not using light).

General comment: there was a typo in Table 1 and in text, selectivity for CO in PEC conditions is 97% and not 99%, we apologize to the reviewer. It has been corrected.

REVIEWERS' COMMENTS:

Reviewer #1 (Remarks to the Author):

From their response to the comments, I can see that our concerns have been dealt very well. The work has been improved lot. So it can be published as it is now.

Reviewer #3 (Remarks to the Author):

The authors adequately answered comments. The edits to the manuscript help clarify the discussion and remove ambiguity.

Reply to reviewers MS: NCOMMS-20-11404A

Reviewer #1 (Remarks to the Author):

From their response to the comments, I can see that our concerns have been dealt very well. The work has been improved lot. So it can be published as it is now.

We thank the reviewer for positive evaluation of the MS.

Reviewer #3 (Remarks to the Author):

The authors adequately answered comments. The edits to the manuscript help clarify the discussion and remove ambiguity.

We thank the reviewer for positive evaluation of the MS.
